# Clinical Correlates of Insulin Resistance in Chronic Schizophrenia: Relationship to Negative Symptoms

**DOI:** 10.3389/fpsyt.2019.00251

**Published:** 2019-04-23

**Authors:** Virawudh Soontornniyomkij, Ellen E. Lee, Hua Jin, Averria Sirkin Martin, Rebecca E. Daly, Jinyuan Liu, Xin M. Tu, Lisa Todd Eyler, Dilip V. Jeste

**Affiliations:** ^1^Department of Psychiatry, University of California San Diego, La Jolla, CA, United States; ^2^Sam and Rose Stein Institute for Research on Aging, University of California San Diego, La Jolla, CA, United States; ^3^Department of Family Medicine and Public Health, University of California San Diego, La Jolla, CA, United States; ^4^Desert-Pacific Mental Illness Research Education and Clinical Center, Veterans Affairs San Diego Healthcare System, San Diego, CA, United States; ^5^Center for Healthy Aging, University of California San Diego, La Jolla, CA, United States; ^6^Department of Neurosciences, University of California San Diego, La Jolla, CA, United States

**Keywords:** body mass index, cognitive function, depression, hemoglobin A1c, psychosis, antipsychotics

## Abstract

Higher prevalence of physical comorbidity and premature mortality in persons with schizophrenia (PwS) results primarily from heightened cardiovascular and metabolic risks. The literature suggests that insulin resistance precedes the development of obesity, smoking, and use of antipsychotic medications, although these likely play a compounding role later in the course of the disorder. It is thus important to discover the clinical characteristics of PwS with high insulin resistance, as these individuals may represent an etiopathologically distinct subgroup with a distinct course and treatment needs. We conducted a cross-sectional study and compared insulin resistance between 145 PwS and 140 nonpsychiatric comparison (NC) participants, similar in age, sex, and race distribution. In addition, we examined correlates of insulin resistance in PwS. As expected, PwS had higher levels of insulin resistance [Homeostatic Model Assessment of Insulin Resistance (HOMA-IR)] and body mass index (BMI) compared to the NC participants. HOMA-IR in the PwS was most associated with negative symptoms, BMI, and non-White race/ethnicity. The mechanistic relationships between insulin resistance and negative symptoms in schizophrenia patients warrant further investigation, and future studies should examine outcomes of PwS with this cluster of physical and mental symptoms and determine how management of insulin resistance might improve health of these individuals.

## Introduction

The mortality gap between persons with schizophrenia (PwS) and the general population is driven, to a large extent, by cardiovascular disease-related deaths (1). Metabolic abnormalities and metabolic syndrome, which are highly prevalent among PwS, are strong independent predictors of cardiovascular-related mortality (2–4). Compared with healthy subjects, schizophrenia patients are more likely to have metabolic comorbidities, including obesity, impaired glucose homeostasis, and metabolic syndrome (5–8). Thus, understanding individual differences among PwS in the degree of metabolic dysfunction could help to identify subgroups who may have an accelerated course of aging and who may be good candidates for preventative measures to reduce morbidity and mortality.

An important contributor to metabolic comorbidities is insulin resistance, which has been shown to precede the cumulative effects of antipsychotic medications, sedentary behaviors, unhealthy diet, and smoking (9–15). In a systematic review and meta-analysis by Pillinger et al. (16), impaired glucose homeostasis, including insulin resistance (Homeostatic Model Assessment of Insulin Resistance or HOMA-IR), was documented in antipsychotic-naive patients with first-episode schizophrenia. Several studies have shown elevated HOMA-IR levels in antipsychotic-naive patients with first-episode psychosis compared with age- and gender-matched controls with similar mean BMI values (17–21). Thus, altered insulin resistance may be part of a whole-body syndrome that manifests in the behavioral disorder of schizophrenia. Consistent with this view is growing evidence that schizophrenia phenotypes may share overlapping genetic loci with metabolic phenotypes (22, 23), and a Mendelian randomization study showing specifically that a genetic predisposition to higher fasting plasma insulin levels is causally linked to an increased risk of schizophrenia (24).

Little is known about the specific demographic and clinical features that characterize PwS who have elevated insulin resistance. Most studies to date have focused on correlates of more general metabolic abnormalities, such as metabolic syndrome and diabetes mellitus (DM). A study by Sicras-Mainar et al. (25) showed that, among 1,120 schizophrenia outpatients on antipsychotic treatment, the prevalence of metabolic syndrome was significantly higher in the patients with one or more negative symptoms (43.9%) than in those without any negative symptoms (34.9%). In a meta-analysis of 12 studies, Bora et al. (26) reported that metabolic syndrome and DM were both associated with more severe cognitive impairment. A focus on the correlates of insulin resistance, which seems to be among the earliest metabolic abnormalities and perhaps intrinsically linked to the pathophysiology of the disorder, may reveal a distinct subgroup of PwS who may have a unique course and could respond differently to treatments.

Our group has examined relationships between metabolic abnormalities and antipsychotic use. One study randomized middle-aged and older patients to one of four atypical antipsychotic medications and tracked metabolic and other adverse outcomes in middle-aged and older adults with schizophrenia and other psychiatric illnesses over 2 years (27). It showed that all the commonly used atypical antipsychotics carried similar high risk of metabolic pathology. The current study is a naturalistic evaluation of the association of other clinical factors (in addition to antipsychotic medication use) possibly related to metabolic dysfunction in a broader age group of PwS, using a multivariate approach. This type of information has considerable potential value for developing targeted interventions to reduce insulin resistance in PwS. This study presents findings from a large sample with extensive clinical phenotyping—allowing us to examine the relationships of insulin resistance with a number of key psychopathological, cognitive, and functioning measures, using multivariate methods to assess the relative contributions of different factors to metabolic abnormalities. PwS often have many risk factors for metabolic abnormalities: lifestyle habits, medications, and underlying biological mechanisms including inflammation and oxidative stress. Treatment and prevention of metabolic abnormalities in PwS can be particularly challenging due to these multiple risk factors (28–32). This study aims to better understand the factors most highly associated with metabolic dysfunction in this high-risk group.

In the present cross-sectional study, we aimed to compare insulin resistance between adults with chronic schizophrenia and nonpsychiatric comparison (NC) subjects, with similar age, sex, and race/ethnicity distribution. Furthermore, within the PwS we examined the relationships between insulin resistance and psychopathology, cognition, and everyday functioning. We performed targeted statistical analyses using the least absolute shrinkage and selection operator (LASSO) method to see what group of factors were most associated with insulin resistance in PwS. These findings will help us better understand what patient characteristics and clinical factors would predict response to interventions to prevent or treat diabetes in PwS. This knowledge would help develop preventive and therapeutic interventions to reduce insulin resistance in schizophrenia and thereby reduce some of the excess morbidity and mortality that characterizes this serious mental and physical illness. We hypothesized that PwS would have worse insulin resistance compared to NCs. We also hypothesized that HOMA-IR would be related to worse psychopathology, cognitive performance, and everyday functioning within the schizophrenia group, even while controlling for BMI and other clinical covariates.

## Materials and Methods

### Study Participants

We recruited 145 persons with chronic schizophrenia receiving outpatient psychiatric treatment and 140 NCs from the greater San Diego area, ranging in age from 26 to 65 years. Analyses on subsets of these data have been reported previously (33–37). The Human Research Protections Program at University of California San Diego approved the study protocol. All participants provided written informed consent prior to participation. The Structured Clinical Interview for *the Diagnostic and Statistical Manual of Mental Disorders, Fourth Edition−Text Revision* (DSM-IV-TR, SCID) was used to determine schizophrenia diagnosis (38). The Mini-International Neuropsychiatric Interview (MINI) (39) was used to screen NC participants who did not have major neuropsychiatric disorders. Exclusion criteria were as follows: 1) other current DSM-IV-TR Axis I diagnoses; 2) alcohol or other substance use disorders (except nicotine or caffeine) within prior 3 months; 3) diagnosis of dementia, intellectual disability disorders, or other major neurological disorders; and 4) any medical disability affecting the ability to complete study procedures. Trained research staff interviewed the study participants, reviewed available medical records, and administered standardized physical and psychological assessments in person.

### Sociodemographic Variables

Demographic features included in the present analysis included age, sex, race/ethnicity, education, and cigarette smoking (**Table 1**).

**Table 1:** Comparison of study participants with and without schizophrenia.

	Nonpsychiatric comparison group			Schizophrenia group			t or χ^2^	df	p	Cohen’s d
	N	Mean	SD	N	Mean	SD				
Sociodemographic variables										
Age (years)	140	48.7	11.2	145	48.3	10.1	0.27	283	0.79	0.03
Sex (% female)	140	54		145	46		1.85	1	0.17	
Race/ethnicity	140			145			4.93	2	0.09	
Non-Hispanic White (%)		58			45					
Hispanic (%)		24			30					
Other (%)		18			25					
Education (years)	140	14.5	2.3	145	12.4	2.3	8.16	283	<0.001	0.97
Cigarette use (packs per day)	140	0.02	0.09	145	0.38	0.50	−8.49	283	<0.001	−1.01
Psychopathology										
Antipsychotic daily dose^1^				145	1.85	1.56				
Duration of illness (years)				142	25.0	11.1				
Positive symptoms (SAPS)				145	6.52	4.20				
Negative symptoms (SANS)				145	7.21	4.35				
Depression (PHQ-9)	130	1.95	2.81	138	7.80	6.59	−9.37	266	<0.001	−1.16
Perceived stress (PSS)	130	10.9	6.04	141	18.7	6.20	−10.5	269	<0.001	−1.28
Cognitive performance, quality of life, and everyday functioning										
Global functioning (TICS-M)	138	37.4	4.22	141	31.0	6.02	10.2	277	<0.001	1.23
Executive functioning (D-KEFS)	140	0.39	0.61	145	−0.57	0.75	11.8	283	<0.001	1.40
Quality of life (SF-36)	131	54.7	5.83	141	42.8	11.26	10.8	270	<0.001	1.32
Everyday functioning (UPSA-B)	140	84.2	9.9	139	67.7	18.1	9.50	277	<0.001	1.14
Metabolic measures										
Body mass index	139	27.6	6.64	140	32.2	7.35	−5.38	277	<0.001	−0.64
Insulin resistance (HOMA-IR)	126	1.74	1.57	134	3.73	5.07	−4.66	258	<0.001	−0.58

For all measures (except the depression and perceived stress), lower scores suggest worse functioning. D-KEFS, Delis-Kaplan Executive Function System (49, 50); HOMA-IR, Homeostatic Model Assessment of Insulin Resistance (56, 57); PHQ-9, Patient Health Questionnaire-9; measure of depression (43); PSS, Perceived Stress Scale; measure of perceived stress (44); SAPS, Scale for the Assessment of Positive Symptoms; SANS, Scale for the Assessment of Negative Symptoms; SF-36, Medical Outcomes Survey—Short Form 36; measure of mental and physical functioning (61); TICS-M, Telephone Interview for Cognitive Status—Modified (45, 46); UPSA-B, UCSD Performance-Based Assessment-Brief (54, 55).

1 Antipsychotic medication daily dosages were converted to WHO average daily doses based on published standards (40).

### Psychopathology

Dose equivalents of antipsychotics were based on the defined daily dose (DDD; the assumed average maintenance dose per day for a drug used for its main indication in adults) presented by the World Health Organization Collaborating Centre for Drug Statistics and Methodology (40). Duration of illness was assessed for all PwS.

Positive symptoms were measured by using the Scale for the Assessment of Positive Symptoms (SAPS) (41). Negative symptoms were measured by using the Scale for the Assessment of Negative Symptoms (SANS) (41, 42).

Depressive symptoms were assessed with the nine-item Patient Health Questionnaire (PHQ-9, total score range 0–27) (43). Perceived stress was measured with the 10-item Perceived Stress Scale (PSS-10, total score range 0–40); and higher scores indicated more severe perceived stress (44).

### Cognitive Performance, Quality of Life, and Everyday Functioning

All cognitive testing was done in face-to-face interviews. Global cognitive functioning was evaluated with the Telephone Interview for Cognitive Status−Modified (TICS-M, 12-item, total score range 0−50), performed during an in-person assessment using the phone script (45–48). TICS-M has been shown to have high sensitivity (82.4%) and specificity (87.0%) for detecting amnestic mild cognitive impairment in community-dwelling older adults (46). Executive functioning was assessed with selected subsets of the Delis-Kaplan Executive Function System (D-KEFS) (49, 50), including Trail Making Letter-Number Sequencing, Color Word Inhibition (Switching), and Letter Fluency (FAS total). We used standardized z-scores from these D-KEFS subtests in order to create a composite score of executive functioning, as previously described by Palmer et al. (51). Mental health-related quality of life was measured with the 36-item Short Form Health Survey, Mental Component Summary (SF-36 MCS) (52, 53). UCSD Performance-Based Assessment-Brief (UPSA-B) is a test of everyday functioning across a variety of community-based tasks (54, 55). In all these measures, higher scores indicated better functioning.

### Metabolic Measures

The body mass index [BMI = bodyweight (kg)/height (m)^2^] was also assessed based on standard measurements.

Fasting venous blood was used for biochemical assays including plasma glucose (mmol/L) and plasma insulin (mU/L). Both assays were conducted in the Altman Clinical and Translational Research Institute (ACTRI) laboratory at University of California San Diego. The level of insulin resistance was estimated with the homeostatic model assessment of insulin resistance {HOMA-IR = [fasting plasma insulin (mU/L) × fasting plasma glucose (mmol/L)]/22.5, giving normal HOMA-IR of 1} (56, 57). HOMA-IR, a steady-state basal test, was validated as a surrogate marker of insulin resistance with reference to standard stimulated-state tests of insulin resistance such as the hyperinsulinemic-euglycemic clamp (57).

### Statistical Analysis

All variables were assessed for violation of distribution assumptions (skew and kurtosis) and, if appropriate, were log-transformed. The schizophrenia and NC groups were compared using independent samples t-test (continuous variables) or Spearman’s rho (categorical variables).

In the PwS and NC groups, pairwise associations of insulin resistance to clinical, medical, and psychological (continuous) variables were assessed with Spearman’s rho. The null of common Spearman’s correlation between the schizophrenia and NC groups was tested by comparing two independent Spearman’s correlations.

We performed multiple regression analyses, aided by LASSO variable selection, to identify the best multivariable model for HOMA-IR. In the multiple regression analysis, regression coefficients were made commensurate by standardizing each variable. Independent variables were ranked by the order in which they entered the LASSO regression. LASSO overcomes various limitations of classic variable selection procedures such as multicollinearity to provide reliable selection of independent variables (58). Independent variables selected by LASSO were entered into the linear model for further trimming using backward elimination, as univariate analysis may also miss significant predictors, and such models may be biased (59). Multicollinearity of the independent variables was assessed using variance inflation factor (VIF) (60).

For all analyses, unadjusted two-tailed *p*-values were considered significant at *p* < 0.05. Significance was defined as Type I error alpha = 0.05 (two-tailed) for all analyses, and false discovery rate (FDR) was used to account for multiple comparisons to ensure overall Type 1 error at alpha = 0.05. The statistical analyses were conducted using the IBM SPSS Version 25 (IBM Corp., Armonk, New York, USA) and R.

## Results

### Group Comparisons

Characteristics of the schizophrenia and NC groups and group differences in the measured variables are presented in **Table 1**. The two groups were similar in the distribution of age, sex, and race/ethnicity, although PwS had fewer years of education and longer duration of cigarette smoking (*p* < 0.001, both). As expected, the PwS endorsed worse depressive symptoms and perceived stress and had worse cognitive functioning, quality of life, and everyday functioning (*p* < 0.0005, all).

The schizophrenia group had higher levels of insulin resistance (HOMA-IR, t_258_ = −4.66, *p* < 0.001); in addition, BMI was higher (t_277_ = −5.38, p < 0.001). In terms of diabetes treatment, 6% of NCs and 23% of PwS reported current use of diabetes medications. HOMA-IR levels were significantly higher among the PwS on diabetes medications, compared to those not taking medications [5.67 (SD = 6.0) vs. 3.12 (SD = 4.6), t47.0 = −2.75, *p* = 0.009].

### Pairwise Correlates of Insulin Resistance

**Figure 1:**
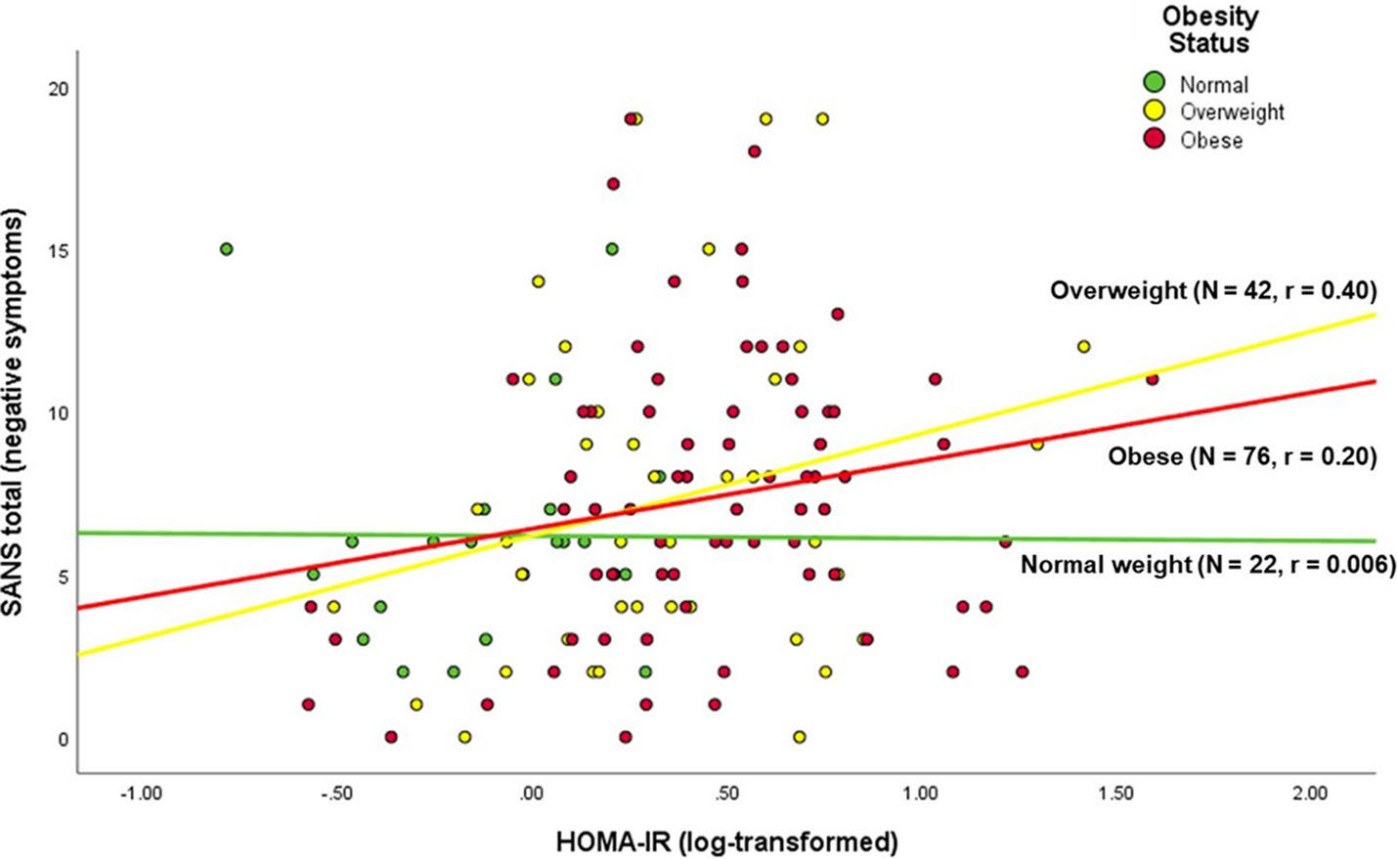
Correlation of negative symptoms with insulin resistance in persons with schizophrenia, stratified by BMI status.

Among the PwS, worse insulin resistance (HOMA-IR) was associated with younger age, higher BMI, worse negative symptoms, worse global cognitive performance, worse quality of life, and worse everyday functioning (**Table 2**, **Figure 1**). Antipsychotic dose, duration of illness, and positive symptoms were not associated with HOMA-IR within the PwS.

**Table 2:** Spearman’s correlations of insulin resistance (HOMA-IR) in participants with and without schizophrenia.

	Nonpsychiatric comparison group			Schizophrenia group			Test for equal correlations	
	N	rho	p	N	rho	p	*z*	*p*
Sociodemographic variables								
Age (years)	126	0.10	0.29	134	−0.18	0.04	2.25	0.02
Education (years)	126	−0.16	0.08	134	−0.04	0.62	−0.97	0.33
Cigarette use (packs per day)	126	0.12	0.19	134	−0.16	0.06	2.25	0.02
Psychopathology								
Antipsychotic daily dose^1^				134	0.07	0.42		
Duration of illness (years)				131	−0.06	0.49		
Body mass index	126	0.55	<0.001	132	0.53	<0.001	0.22	0.83
Positive symptoms (SAPS)				134	0.04	0.69		
Negative symptoms (SANS)				134	0.23	0.007		
Depression (PHQ-9)	117	0.15	0.10	128	0.17	0.06	−0.16	0.87
Perceived stress (PSS)	116	0.18	0.05	131	0.15	0.09	0.24	0.81
Cognitive performance, quality of life, and everyday functioning								
Global functioning (TICS-M)	125	−0.35	<0.001	130	−0.28	0.02	−0.61	0.54
Executive functioning (D-KEFS)	126	−0.29	0.001	134	−0.06	0.46	−1.90	0.06
Quality of life (SF-36)	117	−0.15	0.11	131	−0.23	0.01	0.64	0.52
Everyday functioning (UPSA-B)	126	−0.13	0.16	130	−0.21	0.02	0.65	0.52

For all measures (except the depression and perceived stress), lower scores suggest worse functioning. D-KEFS, Delis-Kaplan Executive Function System (49, 50); HOMA-IR, Homeostatic Model Assessment of Insulin Resistance (56, 57); PHQ-9, Patient Health Questionnaire-9; measure of depression (43); PSS, Perceived Stress Scale; measure of perceived stress (44); SAPS, Scale for the Assessment of Positive Symptoms; SANS, Scale for the Assessment of Negative Symptoms; SF-36, Medical Outcomes Survey—Short Form 36; measure of mental and physical functioning (61); TICS-M, Telephone Interview for Cognitive Status—Modified (45, 46); UPSA-B, UCSD Performance-Based Assessment-Brief (54, 55).

1 Antipsychotic medication daily dosages were converted to WHO average daily doses based on published standards (40).

### Regression Analyses

The best-fit regression model for HOMA-IR had the following factors emerge as significantly associated with higher HOMA-IR: worse negative symptoms, higher BMI, and non-White race/ethnicity (**Table 3**). Collinearity was minimal with VIF < 1.2 for all variables.

**Table 3:** Best-fit general linear models of insulin resistance within persons with schizophrenia (N = 145).

Variable	B	SE	FDR-adjusted *p*
**Race/ethnicity** (Hispanic)	0.18	0.09	0.05
**Race/ethnicity** (other)	0.19	0.09	0.05
**Body mass index**	0.02	0.005	<0.001
**Negative symptoms (SANS)**	0.02	0.008	0.01

SANS, Scale for the Assessment of Negative Symptoms.

## Discussion

Consistent with our hypothesis, we found that insulin resistance was worse in PwS compared to the NC group. We also found that HOMA-IR was related to several variables when considered individually, but the best-fit linear model found non-White race/ethnicity, higher BMI, and worse negative symptoms to be associated with higher HOMA-IR levels in PwS. Positive symptoms, antipsychotic dosage, and duration of illness were not associated with HOMA-IR levels in the PwS. Several measures that were significantly associated individually with HOMA-IR, such as age, quality of life, cognitive performance, and everyday functioning, did not emerge in the multivariate models.

Similar to other published studies (5–8), the sample of PwS studied in the present study had worse psychopathology, cognitive performance, and everyday functioning, as well as higher HOMA-IR and BMI compared with NCs of similar age, sex, and race/ethnicity distribution (26, 62–67). Thus, the findings presented here are likely generalizable to other groups of adults with chronic schizophrenia.

We found an association of insulin resistance (HOMA-IR) with negative symptoms (SANS) in a model that also included BMI and race/ethnicity as significant predictors. Previous observational studies reported associations of metabolic comorbidities such as fasting plasma glucose levels (68), DM (26), and metabolic syndrome (25, 26) with specific psychological symptoms in chronic schizophrenia patients. In antipsychotic-naive patients with first-episode schizophrenia, Chen et al. (19) found that patients with impaired glucose tolerance (assessed with the 75-g oral glucose tolerance test, *n* = 43) had more severe negative symptoms [assessed with the Positive and Negative Syndrome Scale (PANSS)] than those without (*n* = 129) after controlling for age and age of illness onset. In contrast, in another study of antipsychotic-naive patients with first-episode schizophrenia, Steiner et al. (20) found no significant correlation between the level of insulin resistance (HOMA-IR) and the severity of negative symptoms (PANSS). However, in this study of acute schizophrenia (20), the level of insulin resistance (HOMA-IR) was relatively low (given normal HOMA-IR of 1) and the sample size was small {median [interquartile range (IQR)] HOMA-IR = 0.72 [0.38, 2.28], *n* = 24}, as compared with those parameters in our present study of chronic schizophrenia (median [IQR] HOMA-IR = 2.08 [1.21, 4.66], *n* = 134). It is conceivable that the relationship between insulin resistance (HOMA-IR) and negative symptoms of schizophrenia is dependent on the chronicity and severity of illness.

PwS with negative symptoms have been shown to have more severe overall psychopathology, worse cognition, poorer every day functioning, and lower physical activity (69, 70). Negative symptoms may impede help-seeking behaviors and healthcare utilization (71). Thus, PwS with higher levels of negative symptoms may have worse lifestyle behaviors resulting in poorer physical health including greater insulin resistance.

The exact biological processes underlying the relationship of negative symptoms with insulin resistance are unclear. However, dysregulated hypothalamic–pituitary–adrenal (HPA) axis responses and dopamine D2 receptor activity are involved in both psychopathology of schizophrenia and insulin resistance (72, 73). Unexpectedly, PwS with significant negative symptoms were reported to have higher HDL cholesterol levels, which may reflect altered lipid metabolism at the neuronal and systemic levels (74), as well as different patterns of cerebral metabolic activity (75). Adjunctive medications that improve metabolic side effects of antipsychotic medications (e.g., topiramate and pioglitazone) have been shown to also improve negative symptoms (76, 77), possibly through the AMPA/KA receptor antagonism and inhibition of NF-κB expression, respectively. Similarly, exercise has been shown to improve negative symptoms, possibly through increasing serum brain-derived neurotrophic factor and insulin-like growth factor-1 levels (78). Thus, there is a biological basis for our findings. The relationships between insulin resistance and negative symptoms warrant further investigation, especially when targeting interventions for metabolic problems and psychopathology for persons with chronic schizophrenia.

The present study had some limitations. Data on exogenous insulin therapy in our study participants were not available, although we did assess current use of diabetes medications. There are multiple possible contributors to metabolic pathology in schizophrenia. These include lifestyle factors such as physical activity, diet, and sedentary behavior, atypical antipsychotics, and biology of schizophrenia itself (37, 79, 80). In this study, we did not assess lifestyle factors in a systematic way and, therefore cannot comment on their role in the association between negative symptoms and insulin resistance. We found higher HOMA-IR levels among the PwS on diabetes medications, compared to PwS not taking medications. The use of HOMA-IR to assess insulin resistance in individuals specifically on insulin therapy warrants further investigation (57, 81). Furthermore, this is an examination of cross-sectional data to examine insulin resistance, which is highly dependent on longitudinal factors—duration of illness, age, exposure to antipsychotic medications, and other issues. Thus, causality cannot be inferred, though we did include certain time-related factors (duration of illness, daily dose of antipsychotic medications) into our multivariate analyses. Finally, the schizophrenia group consisted of outpatients with a chronic and relatively stable course of illness. The results of the present study may not be generalized to antipsychotic-naive patients with first-episode or acute schizophrenia or hospitalized severely ill patients.

In conclusion, we found that PwS who have higher insulin resistance also have worse negative symptoms as well as higher BMI. Thus, efforts to prevent metabolic comorbidities and subsequent cardiovascular disease and death among chronic patients should focus on PwS with this clinical profile. Our cross-sectional study was not able to discern the temporal relationship among these factors. It is possible, for example, that heightened insulin resistance could be the consequence of unhealthy lifestyle secondary to sedentary behaviors associated with greater negative symptoms in the prodrome or throughout the course of illness (8, 68, 82–85). However, it is also possible, given evidence that insulin resistance exists early in the course of the illness and is linked to genetic risk for the disorder, that glucose dysregulation is a part and parcel of the schizophrenia syndrome, at least for some patients. If so, it may be that a subsyndrome exists in those with high negative symptoms, high BMI, and high insulin resistance.

## Author Contributions

VS conducted and interpreted data analyses and wrote the manuscript. EL and HJ interpreted data analyses and revised the manuscript. AM conducted the study procedures, interpreted data analyses, and revised the manuscript. RD managed data and revised the manuscript. JL supervised statistical analyses and revised the manuscript. XT supervised statistical analyses, interpreted data analyses, and revised the manuscript. LE designed the study and revised the manuscript. DJ designed the study, supervised data management, and revised the manuscript. All authors read and accepted the final version of the manuscript.

## Funding

This work was supported by the United States National Institutes of Health (NIH) [grants R01MH094151-01 to DJ (PI), MH019934 to DJ (PI), and Grant **UL1TR001442 of CTSA funding]**, NARSAD Young Investigator grant from the Brain and Behavior Research Foundation (PI: Ellen E. Lee, MD) and by the Stein Institute for Research on Aging at the University of California San Diego. VS was supported by NIH Grant R56 AG059437. **The **content is solely the responsibility of the authors and does not necessarily represent the official views of the NIH.

## Conflict of Interest Statement

The authors declare that the research was conducted in the absence of any commercial or financial relationships that could be construed as a potential conflict of interest.
